# Expanding the Toolbox of Multi-Material Three-Dimensional-Printed Electrochemical Flow Cells Fabricated in a Single Step: Impinging Jet, Mixing and Dilution, and Dual-Electrode Generator–Collector Electrochemical Cells

**DOI:** 10.1021/acselectrochem.5c00444

**Published:** 2026-01-30

**Authors:** Kayla M. Mancini, Enock G. Arthur, Cameron Darvish, Edgar M. Manriquez, Inara Trongone, Glen D. O’Neil

**Affiliations:** † Department of Chemistry and Biochemistry, 8087 Montclair State University, Montclair, New Jersey 07043, United States; ‡ Sokol Institute for Pharmaceutical Life Sciences, Montclair State University, Montclair, New Jersey 07043, United States

**Keywords:** 3D printing, impinging jet electrode, dual-electrode generator−collector

## Abstract

Hydrodynamic electrochemical cells have broad utility in sensing, electrocatalysis, mechanistic studies, and electroanalysis. Here, we report on the expansion of hydrodynamic electrochemical devices that can be fabricated using 3D printing beyond channel flow electrodes to include impinging jet electrodes, a dilution/mixing circuit for in-channel detection of electroactive species, and dual-electrode generator–collector cells. These examples highlight the ability of 3D printing to produce intricate structures with internal voids, enhance analytical functionality, and enable sophisticated operations such as fluid mixing and generation–collection in simple, monolithic devices that do not require alignment, clamping, or assembly. To promote adoption by the wider electrochemical community, we provide printable .stl files as part of the manuscript. Device performance was evaluated in both stagnant and flowing solutions of ferrocene methanol, an outer-sphere redox mediator suitable for characterizing the transport characteristics and electrochemical performance of the devices. All devices exhibited responses consistent with Levich behavior for their respective geometries. The mixing circuit achieved 100-fold dilutions with results in excellent agreement with solutions prepared using volumetric glassware, while the generator–collector cell demonstrated high collection efficiencies under optimized conditions. These results establish 3D printing as a versatile, accessible approach for fabricating hydrodynamic electrochemical devices with advanced capabilities and pave the way for customizable, high-performance devices suitable for electroanalysis, electrocatalysis, and sensing applications.

## Graphical abstract

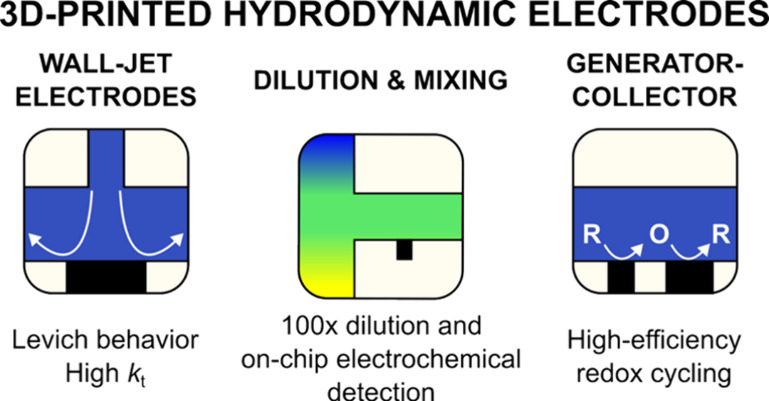

## Introduction

Hydrodynamic electrodes, including rotating disk (RDE), channel-flow (CFE), and impinging jet electrodes (IJE), are widely used in fundamental mechanistic studies, electrocatalysis, and sensing. Hydrodynamic electrodes differ from those based purely on diffusion because convection is used to control reactant/product transport to/from the electrode surface, leading to well-defined mass transport.^1^ Due to higher rates of mass transport, these systems typically achieve a steady-state faster than diffusion-only measurements, which is most apparent when using macroscale electrodes. One of the experimental challenges faced when using hydrodynamic electrochemical methods, especially those where solution is confined in channels like CFEs and IJEs, is the precise fabrication of these devices using conventional machining, which necessitates forming flow cells from multiple components and sealing them together.^2^ In contrast to traditional manufacturing, 3D printing has recently emerged as a promising approach for fabricating electrodes and electrochemical cells because it offers improved design flexibility and the ability to rapidly prototype designs.^3^ 3D printing also offers the advantages of low up-front and operational costs of desktop 3D printers,^4^ inherent ability for customization,^5,6^ ease of fabrication, high performance of the 3D-printed components,^7^ and potential for on-demand fabrication of customized sensors and devices.^8^ While a detailed discussion of the merits, characteristics, and applications of 3D-printed electrodes is beyond the scope of this contribution, there are several recent reviews highlighting advances in 3D printing in electrochemistry.^9–14^

Recently, we demonstrated the ability to fabricate electrochemical flow cells that incorporate both fluid handling and electrochemical sensors in a single step using multi-material 3D printing.^15^ The advantage of using 3D printing for fabricating hydrodynamic flow cells is that the fluid handling components (*e.g.*, the flow body comprising the inlet, outlet, and channel) can be printed together with electrodes, thus eliminating assembly and offering seamless integration of the electrodes with the flow cell. Subsequently, the Kokkinos and Escarpa groups have used similar channel flow electrodes for their studies.^16,17^ In addition to flow cells, 3D printing has also been used by the Amatore/Xu, Banks, and Macpherson groups to produce 3D-printed versions of the RDE and rotating ring-disk electrodes (RRDE), although these were not produced in a single step.^18–20^ The Martin and Banks groups have demonstrated multi-component wall-jet electrodes,^21,22^ and Martin’s group has demonstrated generation–collection experiments using 3D-printed fluidics;^23^ however, these devices all required post-print assembly and, in some cases, incorporation of additional metal electrodes. To date, the majority of single-step 3D-printed flow cells that have been produced are simple channel-flow cells featuring a single working electrode.^15–17^ One exception is from the Escarpa group, who fabricated a multi-electrode, dual channel cell that was modified with Prussian Blue/Au NPs in a print-pause-print strategy for hydrogen peroxide detection.^24^ Therefore, there is a gap in the literature for hydrodynamic electrochemical devices that offer different hydrodynamic properties than CFEs and can perform more complex fluidic and analysis operations.

In this contribution, we expand the toolkit of hydrodynamic electrochemical devices by demonstrating several geometries that are all printed in a single step with a commercial off-the-shelf printer: impinging jet electrodes, a dilution/mixing circuit capable of in-channel detection of electroactive reagents, and dual-electrode generator–collector cells. These devices all take advantage of 3D printing’s ability to fabricate complex, intricate designs in a single step. Moreover, these hydrodynamic electrochemical devices are monolithic and therefore do not require alignment, clamping, or assembly. We selected these particular geometries to (i) highlight that complex designs including internal voids are easily fabricated, (ii) explore alternative cell configurations with interesting hydrodynamic properties, and (iii) demonstrate that complex operations (*e.g.*, fluid mixing and generation–collection) can be performed with devices that can be fabricated in a single step using 3D printing. In order to make these devices more accessible to the broader electrochemical community, we have included printable .stl files in the Supporting Information. We characterized the behavior of each device in stagnant and flowing solutions of ferrocene methanol, an outer-sphere redox mediator, and found that each device behaved according to the Levich equation for the geometry employed. The mixing circuit was able to perform 100-fold dilutions that showed excellent agreement with solutions prepared using volumetric glassware. The generator–collector cell showed collection efficiencies that were comparable to those fabricated using standard techniques (*e.g.*, microfluidics and traditional manufacturing). The results pave the way for customizable, high-performance devices suitable for electroanalysis, electrocatalysis, and sensing applications.

## Experimental Section

### Materials and Solutions

All of the reagents were used as received. Ferrocene methanol (FcMeOH; 97%) was from Acros Organics. Potassium nitrate and sodium hydroxide were from Fisher Scientific and were ACS reagent grade. Solutions were prepared with 18.2 MΩ•cm water purified using a benchtop Millipore Simplicity system. Transparent poly­(lactic acid) (PLA) was purchased from Ultimaker and commercial conductive PLA containing carbon black was from ProtoPasta. ProtoPasta contains about ∼24 ± 2 wt % carbon black and the balance was PLA and has a volume resistivity of 30 Ω•cm (in plane).^7^ Conductive silver paint was from Pelco and copper wire was from McMaster Carr.

### Design and Fabrication of 3D-Printed Devices

All components were designed in Autodesk Inventor Pro, which is free for academic users at the time of writing. The flow bodies and electrodes were designed separately and were test fit prior to printing using the Assemblies feature in Autodesk Inventor to ensure that there were no detectable gaps in the two mating components. *.stl* files, which can be directly used with any commercial slicing software, for each device type are available as part of the Supporting Information. The components were exported as .stl files before being assembled and sliced in Cura. Alignment of the parts for each device requires careful attention to detail, as misalignment of the electrodes with the flow bodies can cause the devices to leak and deviate from their ideal responses. Many commercial slicers, including Cura, will automatically align multi-component prints if the prints are designed around a common coordinate system. In other words, the position of each object relative to the origin should be consistent among the parts. The devices were printed on an Ultimaker S3 dual extrusion 3D printer equipped with a 0.4 mm Model AA print core using transparent PLA for the flow bodies and carbon black/PLA for the electrodes. The devices were printed with a 0.1 mm layer height, 3 wall layers (1.2 mm thickness), 12 top & bottom layers (1.2 mm thickness), 70 mm s^–1^ print speed, 195–200°C hot-end temperature, 60°C build plate temperature, a skirt, and a 15 mm (standard) prime tower. The prime tower ensured that the electrodes were not under-extruded after material switching. The flow bodies and electrodes were printed at 20% and 100% infill, respectively. In order to improve adhesion between the glass print bed and the flow cells, a thin layer of UHU Stic glue stick was applied to the glass surface. For devices with small electrodes and electrode gaps, we found that manually leveling the print bed before printing eliminated mixing between the two filaments.

All dimensions described in the manuscript correspond to the dimensions defined by the design file. We characterized the agreement between the design and the printed parts as described in Section S1 and Figure S1 in the Supporting Information. For the sizes of devices used here, the dimensions of the printed parts are within ∼20% of the designed dimension.

Modifiable 3D design files (.ipt) are available to non-commercial users by request to the corresponding author. In addition, .stl versions all of the devices used here (and others from our group) are available on our group Printables.com page: https://www.printables.com/@gdolab_3923699.

#### Impinging Jet Electrodes

A schematic of an impinging jet electrode is shown in Figure 1a and detailed cutaways of the flow device and electrodes are shown in Figure S2 in the supporting information. These electrodes were designed and tested in the wall-jet electrode (WJE) configuration. Unless otherwise noted, wall-jet electrodes were prepared with an electrode diameter of 3 mm and an inlet diameter of 1.5 mm. The height of the flow chamber was 1 mm, which was tall enough to not interfere with the impinging jet profile. In the prototyping phase, we observed significant stringing and sagging of the ceiling of the flow chamber that affected flow in the devices. In response, we added eight thin baffles (0.5 mm) spaced radially within the channel. The baffles were placed so that the inner diameter was larger the outer diameter of the central working electrode. A pressure equilibration chamber, designed to ensure uniform flow within the impinging jet, was placed above the electrodes and was connected to a single outlet channel.

**Figure 1:**
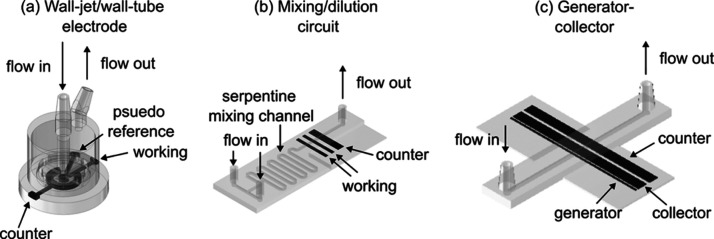
Expanded toolkit of 3D-printed hydrodynamic electrodes. Schematic 3D-printed (a) impinging jet electrode, (b) mixing and dilution circuit with built in electrochemical detection, and (c) dual-electrode generator–collector cell. Photos of example devices can be found in Figure S3 in the Supporting Information.

#### Mixing Circuit

A schematic of the mixing circuit is shown in Figure 1b. The mixing circuit contained two inlets, each 2 mm in diameter, that lead into channels with a width (*w*) and height (2*h*) equal to 0.1 and 0.075 cm, respectively. The two channels meet at a T-junction, which flowed into a serpentine channel that contained nine 180° turns before forming a straight channel that flows over three band electrodes. The first two band electrodes have a length (*x*_e_) of 0.12 cm and the third electrode has a length of 0.3 cm. The electrodes were placed 0.5 cm from the final turn in the mixer, which was a sufficient entrance length for laminar flow to be fully developed before the electrodes for flow rates less than 0.033 mL s^–1^. In these experiments, the first band electrode was used as the working electrode, with the middle electrode printed for redundancy in the case of a bad electrical connection, and the third band electrode was used as a counter electrode. While it was not used herein, the three electrodes could be used to form an on-chip three electrode cell with the upstream electrode serving as a reference, the middle electrode as a working, and the downstream electrode as a counter.

#### Generator–Collector Cells

A schematic of an example generator–collector cell is shown in Figure 1c. The devices featured a 5 cm long channel with *w* = 0.12 cm and 2*h* = 0.05 cm. The leading edge of the generator electrode was placed 2.4 cm from the center of the inlet. The hydrodynamic entrance length for this device was less than 1 cm for all of the flow rates used in these experiments. These cells were challenging to print because of the small size of the generator electrode and the gap between the electrodes. We first performed an experiment to determine the minimum electrode width and electrode spacing for our printer and print settings. In this experiment, we designed and printed a device that had band electrodes ranging from 0.01–0.1 cm (in 0.01 cm increments) and spacings that ranged from 0.005–0.1 cm. A schematic of the device is shown in Figure S4 in the supporting information. In our 3D printer configuration, the smallest electrode width achieved was 0.05 cm and the smallest spacing was 0.05 cm, which is consistent with the hot-end used for fabrication. There is scope for improving the resolution by employing a smaller diameter hot-end, although in our experience these are clogged more easily with composite filaments. During the prints, we observed that at the end of the electrodes, where the print head changes direction, there was significant broadening of the electrodes leading to overlap between adjacent electrodes when the spacing was close together. To combat this, we extended the print and cut the device perpendicular to the electrodes ∼1 cm from the flow body.

### Electrochemical Measurements

Electrical connection to the electrodes was made by fixing a copper wire to the electrode contact traces outside of the flow cell using silver epoxy after briefly sanding the contact surface with 600 grit paper. Because of the close spacing between electrodes in the generator collector cell, it was challenging to make a selective electrical connection with all of the contacts on one side of the device. To combat this, we made the connection to the generator and counter electrode on one “wing” of the device and the electrical connection to the collector electrode on the opposite wing (Figure S5a). A small amount of Araldite two-part epoxy was applied to strengthen the electrical contacts for all of the devices. The measurements using the WJEs and mixing circuits were performed in a three-electrode cell; the measurements using the dual-electrode generator–collector were performed in a four-electrode cell with two working electrodes. Unless otherwise noted, a commercial SCE reference electrode was placed in the outlet reservoir. All measurements were made using a CHI 660C, 760E, or 760F (bi)­potentiostat controlled by a PC. Figure S5a shows a close-up photograph of a dual-electrode generator–collector cell.

Fluid flow was controlled using a single-barrel (KD Scientific) or dual-barrel (Ossila) syringe pump. Photos of the flow setup for a dual-electrode generator–collector cell is shown in Figure S5b. Luer-lock syringes were 20 mL and sourced from BD Plastics. Fluid connections to the devices were made by pressure fitting a 1/16″ Luer-lock barb onto the inlets and outlets with the aid of a few layers of Parafilm. The connection between the syringe barrel and flow cells was made using 1/16” silicone tubing.

The electrodes were activated using the procedure developed by Richter et al., where +1.4 V was applied for 200 s, followed by a −1 V for 200 s in 0.5 M NaOH solution flowing at 0.0167 mL s^–1^.^26^ SEM images of as-printed electrode surfaces are shown in Figure S6, showing texture to the electrodes on the ∼tens of micrometer scale. The effect of activation on the hydrodynamic response of channel flow electrodes is shown in Figure S8. The hydrodynamic slope is ∼25% larger after activation, suggesting changes to the electrode size and/or channel geometry. The PLA used for fabrication is not stable in 0.5 M NaOH for long periods of time and some devices (estimated as ∼10% of more than one hundred tested) failed mechanically, typically at the inlet or outlet ports, during or after the activation protocol. We note that other protocols are available for activating channel flow electrodes.^17^

## Results and Discussion

### Impinging Jet Electrochemical Cells

Impinging jet (*i.e.*, wall-jet and wall-tube) electrodes feature a jet of solution that flows axially towards the center of a disk-shaped electrode and radially over the surface of the electrode after contact (Figure 2a).^27^ These types of electrodes are widely used as amperometric detectors for chromatography^21,22^ and are readily coupled with spectroscopic techniques.^28^ For wall-jet electrodes, the radius of the impinging jet, *r*_inlet_, is smaller than the radius of the electrode, *r*_electrode_, while for wall-tube electrodes, *r*_inlet_ > *r*_electrode_. We are interested in developing impinging jet electrodes because the mass transfer coefficient, *k*_t_, is more sensitive to the flow rate compared to channel flow electrodes (see comparisons in Section S2 and Table S1 in the Supporting Information). Higher *k*_t_ values are advantageous in analytical sensing platforms as well as for kinetic studies. In addition, the design of these types of electrodes is significantly more complex than a simple channel flow electrode, which showcases the capabilities of 3D printing, and in particular multi-material 3D printing, compared with traditional manufacturing.

**Figure 2:**
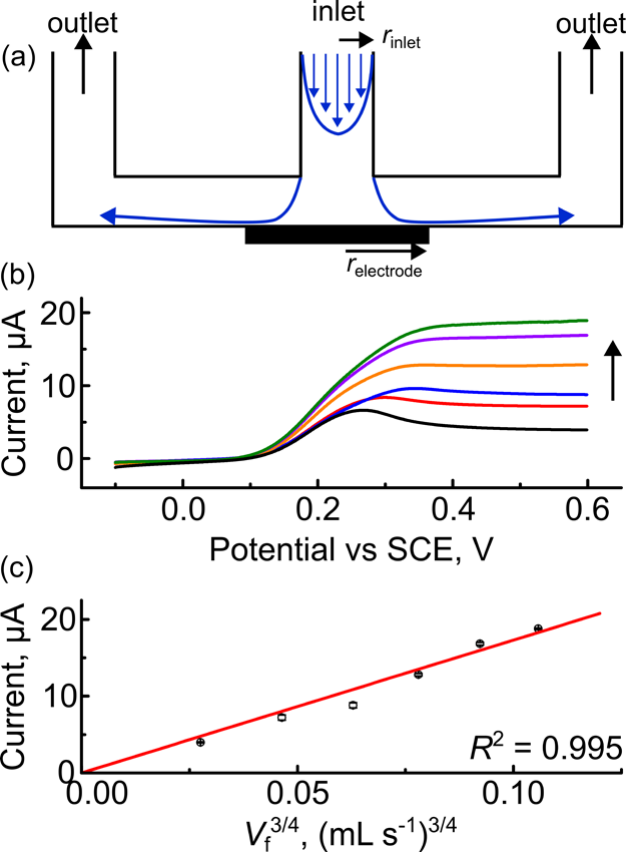
Impinging jet electrodes are easily fabricated using multi-material 3D printing and have a limiting current versus flow rate relationship consistent with the Levich equation. (a) Schematic of the impinging jet electrode; (b) average (*n* = 5) LSVs of the oxidation of 1 mM FcMeOH in 0.1 M KNO_3_ at 0.05 V s^–1^ at flow rates ranging from 0.008 (black trace) to 0.5 (green trace) mL s^–1^. The arrow shows increasing flow rates; (c) *i*_lim_ versus *V*_f_^3/4^ for the data shown in part (b).

The limiting current, *i*_lim_, of a wall-jet electrode is described by^29^

$$i_{\lim}=\frac{1.597nFk_{c}r_{electrode}^{3/4}D^{2/3}V_{f}^{3/4}c^{*}}{v^{5/12}r_{inlet}^{1/2}}$$ 1

where *n* is the number of electrons transferred, *F* is faraday’s constant (= 96485 C mol^–1^), *k*_c_ is a constant (=0.9) that describes the momentum flux, *v* is the kinematic viscosity of solution (in cm^2^ s^–1^), *D* is the diffusion coefficient (in cm^2^ s^–1^), *V*_f_ is the volumetric flow rate (in cm^3^ s^–1^), and c* is the bulk concentration (in mol cm^–3^) of redox species. The derivation of eq 1 assumes that the height of the device does not impact the wall-jet fluid dynamics and that the flow is laminar.

We designed wall-jet electrodes with *r*_inlet_ = 0.1 cm, *r*_electrode_ = 0.15 cm. The flow chamber height (*i.e.*, the height of the ceiling above the electrode) was 0.1 cm for both devices. A disk-shaped working electrode was placed directly underneath the inlet, a C-shaped truncated ring electrode was placed outside of the disk, and a square third electrode, which could be used as a quasi-reference electrode, was placed next to the working electrode (Figure S2c). One important consideration for operating wall-jet electrodes is to ensure a uniform pressure drop within the cell so that solution travels outward radially from the center of the electrode surface towards the outlets. In order to maintain uniform hydrodynamics within the cell, the single inlet empties into four slotted outlets (0.6 cm i.d., 0.7 cm o.d.), which are evenly spaced in the cell at positions in line with the circumference of the working electrode (Figure S2a). In order to maintain an even pressure drop between the inlet and outlets, all four outlet slots open into a chamber above the electrodes to form a single waste outlet (Figure S2b). This design was made possible by the ability of 3D printers to fabricate structures with internal voids, and would have been impossible to fabricate using high-precision subtractive manufacturing techniques without bonding multiple components together.

To assess electrochemical behavior of the printed devices, we performed cyclic (CV) and linear sweep voltammetry (LSV) for the oxidation of 1 mM FcMeOH in 0.1 M KNO_3_ at 0.05 V s^–1^ collected at flow rates ranging from 0–0.05 cm^3^ s^–1^ at a scan rate of 0.05 V s^–1^. Under stagnant conditions (*V*_f_ = 0 cm^3^ s^–1^), the CV shows quasi-reversible electron transfer with Δ*E*p ≈ 130 mV (Figure S9). We found close agreement between the experimental peak current (*i*p = 6.0 μA) with the expected peak current for a 0.15 cm radius disk electrode calculated using the Randles equation for a quasi-reversible redox reaction (*i*p ≈ 5.8 μA; details in supporting information). This suggests that the entire disk is behaving as a uniform electrode surface caused by diffusional overlap to the isolated carbon particles on the surface.^7^ For flowing solutions, we observed that the limiting current increases as the flow rate increases. At the lowest flow rates, the LSVs showed small peaks, which suggests that for this combination of scan rate and flow rates, the mass transfer behavior is a mixed diffusion/convection response near the peak potentials. At higher potentials (>0.45 V), the traces reach a steady-state limiting value. Figure 2c shows a plot of *i*_lim_ vs. *V*_f_^3/4^ where the gradient is linear (*R* ^2^ = 0.995). The gradient of the best fit line (173 ± 5 μA mL^3/4^ s^–3/4^) showed excellent agreement with the value calculated using eq 1 (172 μA mL^3/4^ s^–3/4^), assuming the following values: *n* = 1, *k*_c_ = 0.9, *r*_electrode_ = 0.15 cm, *v* = 8.8×10^–3^ cm^2^ s^–1^, *D* = 7.8×10^–5^ cm^2^ s^–1^, *r*_inlet_ = 0.075 cm, and c* = 5.0×10^–7^ mol cm^–3^. Interestingly, these devices show much better agreement to theory than our previous channel flow electrodes.^15^

The mass transfer coefficient, *kt*, for a wall-jet electrode is described by

$$k_{t}=\frac{i_{\lim}}{nFAc^{*}}=\frac{0.458D^{2/3}V_{f}^{3/4}}{v^{5/12}r_{inlet}^{1/2}r_{electrode}^{5/4}}$$ 2

Where all of the variables have the same meaning as eq 1. For the device in Figure 2b, the *k*_t_ was calculated from the limiting current to be 0.003 cm s^–1^ at the highest flow rate that our pump can deliver (= 0.05 mL s^–1^). The experimentally determined *k*_t_ is in reasonable agreement with the value calculated using eq 2 (= 0.005 cm s^–1^; details in the supporting information Section S2). At the same flow rates, a channel flow electrode with dimensions of *w* = 0.47 cm, *x*_e_ = 0.15 cm, and 2*h* = 0.1 cm has an estimated *k*_t_ of only 0.00026 cm s^–1^ (Table S1). Note that in this comparison, we set *w* and *x*_e_ so that the electrode area would be identical to the WJE with *r*_electrode_ = 0.15 cm. We note that *k*_t_ values as high as 0.5 cm s^–1^ have been observed in the literature for microfabricated CFEs.^30^ However, achieving these high rates required microfabrication, with the devices having dimensions of *x*_e_ = 40 μm, 2*h* = 20 μm, and *w* = 100 μm. Interestingly, increasing the flow rate has a dramatic effect on the WJE but a limited effect on the CFE because of the differences of the current dependence on flow rate (*V*_f_^3/4^ for the WJE and *V*_f_^1/3^ for the CFE). When the flow rate is increased from 0.05 mL s^–1^ to 0.25 mL s^–1^, *k*_t_ is expected to increase from 0.005 to 0.017 cm s^–1^ for the WJE, but only from 0.00026 to 0.00044 cm s^–1^ for the CFE.

To evaluate the performance of the wall-jet electrode at higher flow rates and test their capacity for enhanced mass transfer, we constructed a gravity-fed pump system as described in Section S3. This system was needed because the syringe pumps in our lab have a maximum flow rate of ∼0.05 mL s^–1^. Figure S10a shows representative LSVs for the oxidation of 0.5 mM FcMeOH in 0.1 M KNO_3_ over the range of flow rates from 0.017 to 0.05 mL s^–1^ (acquired with syringe pump) and at ∼4 mL s^–1^ (acquired with gravity pump) using an impinging jet electrode with a 0.2 cm diameter electrode and 0.2 cm inlet. At the lower flow rates, this device showed increasing limiting currents with increasing volumetric flow rates, as expected (Figure S10b). Under the high flow rates of the gravity pump, the devices showed approximately a 10-fold increase in current compared with the highest flow rates achievable with the syringe pump. Using the gravity-fed system, the limiting current was 26.2 ± 0.8 μA, corresponding to a *k*_t_ of ∼0.02 cm s^–1^. We also observed a significant increase in the experimental noise during measurements at high flow rates. We suspect that this is due to the flow is in the intermediate region between Laminar and turbulent flows, with a Reynolds number of ∼1800. To achieve a similar *k*_t_, an RDE would have to rotate at ∼230 Hz (∼13,800 rpm) while a UME would require a radius of 5 μm. One drawback of the WJE is that large volumes of solutions are required for experiments. While these solutions can be recirculated or recycled, it is inconvenient to perform these experiments and they would be impossible to perform if small amounts of sample were available. There is scope for improving *k*_t_ can be further improved upon by decreasing the inlet radius (*e.g.*, by using capillary tubing in the inlet) or decreasing the electrode radius.^31^

### Serpentine Mixing Circuit with Built-in Electrodes

We next consider a fluidic circuit for performing dilution/mixing before downstream electrochemical detection. Dilution and mixing are perhaps the most common sample preparation steps and are necessary for preparing standards, adjusting analyte concentrations in samples, and decreasing matrix effects in complex samples. Typically, these operations are performed by hand using volumetric glassware or calibrated micropipettes and can be laborious if many standards are required. Performing these fundamental lab operations is also possible using hydrodynamic circuits, and there are many designs and options for fluidic on-chip mixing varying widely in complexity.^32,33^ Here, we opted for simplicity, using the inherent roughness of the channel and changes to the fluid path to force rapid diffusional mixing.^34,35^ These devices (Figure 1b) consist of two inlets that merge to form a single serpentine channel with nine turns. After the turns, the channel straightens out and flows over a series of band electrodes. The band electrodes are placed beyond the hydrodynamic entrance length (∼0.5 mm) to ensure laminar flow is established by the time the fluid front flows over the electrodes. This geometry is essentially a channel flow electrode (CFE) with a long serpentine channel preceding the straight channel used for the electrodes.^15^

We first confirmed that the mixing circuit was providing the correct volumes using UV/vis spectroscopy using Fe­(CN)_6_^3–^. We used UV/vis because concentration can be determined from absorbance, *A*, directly using Beer’s Law:

A=εbc 3

where ε is the molar extinction coefficient (=1020 M^–1^ cm^–1^ at 420 nm), *b* is the path length (=1 cm), and *c* is the molar concentration. In these experiments, a 3 mM stock solution of K_3_Fe­(CN)_6_ flowed through one inlet and a 0.1 M KNO_3_ diluent solution flowed through the other. We performed dilutions of the stock solution by varying the *V*_f_ ratio (*V*_f,stock_/*V*_f,diluent_) of the two inlets while maintaining a consistent overall flow (*V*_f,tot_ = 0.016 mL s^–1^ or 0.032 mL s^–1^; approximately 1 or 2 mL min^–1^). In this way, the ratio of *V*_f,stock_/*V*_f,tot_ serves as a dilution factor for the stock solution (Table S2). We collected approximately 10 mL of sample from the outlet and analyzed each using UV/vis. UV/vis spectra of Fe­(CN)_6_^3–^ over the concentration range from ∼0.03–3 mM are presented in Figure 2a, showing an increase in absorbance as the concentration of Fe­(CN)_6_^3–^ increases. We determined the concentration of each solution from the absorbance spectra using Beer’s law and compared is to the concentration expected from the *V*_f_ ratio dilution factors (Table S2). Figure 3b shows a linear relationship (*R* ^2^ > 0.9999) between observed and expected concentration with a slope equal to unity (1.000 ± 0.002 and 1.003 ± 0.002) for 0.016 and 0.032 mL s^–1^ flow rates, respectively). These experiments demonstrate that the simple mixing circuit performs quantitative mixing; however, a drawback of these measurements is that the analysis takes place *ex situ*, giving the solutions significant time to mix by diffusion and convection in the storage bottle.

**Figure 3:**
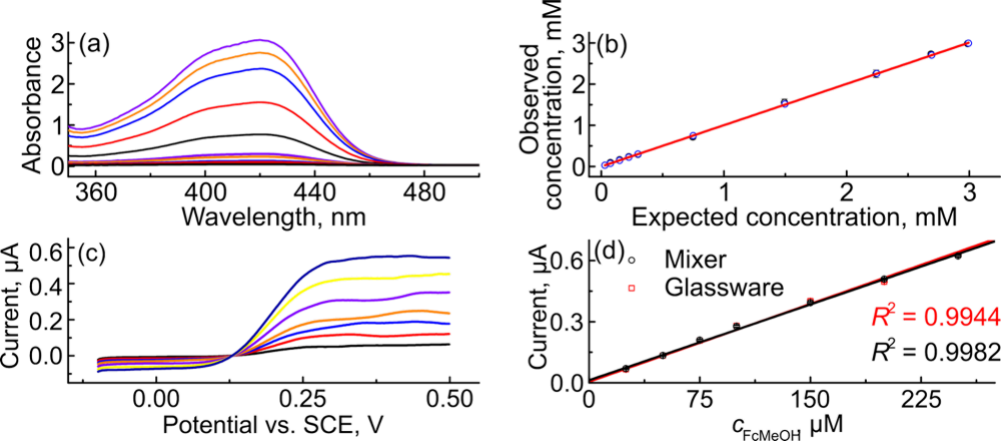
3D-printed mixing/dilution cell is able to deliver 100:1 dilutions and perform electrochemical detection *in situ*. (a) UV/vis spectra of 0–3 mM Fe­(CN)_6_^3–^ solutions collected at different concentrations prepared using a 3D-printed mixer. (b) Comparison plot showing the relationship between the measured concentration and the concentration expected based on dilution factor from the *V*_f_ ratio. (c) LSVs for the oxidation of FcMeOH in 0.1 M KNO_3_ at concentrations ranging from 25 μM to 250 μM. Solutions were prepared using a 250 μM FcMeOH stock solution (also containing 0.1 M KNO_3_) in one inlet and a 0.1 M KNO_3_ solution in the second inlet. (d) Comparison of calibration curves prepared with the mixing circuit (black symbols and line) with standards prepared *ex situ* using volumetric glassware. Scan rate = 0.05 V s^–1^; counter = CB/PLA; reference = SCE.

We next investigated the ability to perform dilution/mixing with built-in electrochemical detection using flowing solutions of FcMeOH. Given that these experiments measure current in a flowing stream, it was first necessary to characterize the mass transport within the device.^15^ The mixing circuit with embedded electrodes is based on a channel-flow electrode configuration. When laminar flow is fully developed and changes in concentration are confined near the electrode, the parabolic flow profile can be linearized using the Lévêque approximation, the limiting current in a CFE is^36^

$$i_{\lim}=\frac{0.925nFc^{*}D^{2/3}V_{f}^{1/3}w^{2/3}x_{e}^{2/3}}{h^{2/3}}$$ 4

where *n*, *F*, c*, *D*, and *V*_f_ have the same definitions as eq 1, and *w*, *x*_e_, and *h* are the channel width, electrode length in the direction of flow, and one-half the channel height, respectively. Figure S11a shows LSVs for the oxidation of 50 μM FcMeOH in 0.1 M KNO_3_ collected at 0.016, 0.032, and 0.048 mL s^–1^. Qualitatively, the results show that as the volumetric flow rate increases, the steady state limiting current increases, as expected. A gradient of the *i*_lim_ vs. *V*_f_^1/3^ plot is linear (*R* ^2^ = 0.9997), and has a value of 0.66 μA s^1/3^ mL^–1/3^, confirming that the flow within the channel is laminar and parabolic. The theoretical response of the electrode calculated using eq 4 was 0.81 μA s^1/3^ mL^–1/3^. We suspect that difference between the predicted slope and the experimental slope is driven by some combination of the channel geometry of the printed part does not matching the .CAD file, the printed surfaces having some roughness that effects the flow profile, or that the electrodes not sitting co-planar with the floor of the channel. We are developing methods for probing the inner geometry of the enclosed channels and will report on those findings in due course.

We next performed dilutions and on-chip detection using FcMeOH as an example molecule. Figure 3c shows LSVs of FcMeOH oxidation collected at 0.05 V s^–1^ and 0.016 mL s^–1^ for solutions containing different concentrations of FcMeOH. In these LSVs, the solutions were prepared on-chip using a 250 μM FcMeOH in 0.1 M KNO_3_ solution as the stock and 0.1 M KNO_3_ as the diluent. The LSVs show an increase in steady-state limiting current with concentration, with some noise caused by fluctuations in flow due to the pump. Figure 3d shows a plot of the limiting current versus FcMeOH concentration over the range from 0 to 250 μM. The black circles are data collected using on-chip mixing/dilution, while the red squares are data collected using standards produced using volumetric glassware. The calibration curves collected using solutions prepared with the mixing device (black) and volumetric glassware (red) show excellent agreement: the gradient of the calibration curve prepared using the on-chip mixer is 2.48 ± 0.04 nA μM^–1^ and the volumetric glassware control was 2.55 ± 0.08 nA μM^–1^. These data confirm that mixing within the device is complete when the solution reaches the electrodes and that on-chip mixing/dilution can be coupled with on-chip detection.

The devices described herein perform comparably well to other examples of fluidic mixers in the literature. For instance, Podunavac et al. developed a device for measuring glucose that included serpentine mixers for pH adjustment and addition of glucose oxidase enzyme.^37^ The device used a commercial screen printed electrode for measuring the glucose concentrations and was fabricated using stereolithography (SLA) 3D printing. Kaufman et al. developed an automated platform for monitoring oxygen.^38^ The authors provided detailed electrochemical characterization of the flow system using a standard redox couple (ferri-/ferrocyanide) showing 10-fold dilutions.

### Dual-Electrode Generator–Collector Flow Cells

Finally, we consider dual-electrode generator–collector cells (DCE), which are conceptually similar to other generator–collector type systems including the rotating ring disk electrode (RRDE)^39^ and the generator–collector modes of scanning electrochemical microscopy (SECM).^40^ In a DCE, shown schematically in Figure 4a, two electrodes are placed at the bottom of a rectangular channel. An upstream generator electrode drives an electrochemical reaction of interest (*e.g.,* R → O + e^–^) at a mass transfer limited rate. The reaction product is carried downstream to the collector electrode, which is biased so that the reverse reaction occurs (*e.g.*, O + e^–^ → R). These systems are widely used in the electrochemical literature and have been studied experimentally and theoretically.^36,41^ Macpherson and co-workers showed that an upstream electrode can locally modify the pH of solutions for improved heavy metal measurements^42^ and sulfide detection.^43^ Crooks and co-workers have used microfluidic versions of these types of cells to characterize electrocatalyst products,^30,44^ titrate thin catalyst films,^45^ and investigate electrochemical self-assembled monolayers.^46^ Earlier, the Compton, Unwin, and other groups used these systems to probe homogeneous chemical reactions between an electrogenerated reactant and solution based product.^47–49^ We were motivated to create a 3D-printable variant of this configuration due to this broad utility.

**Figure 4:**
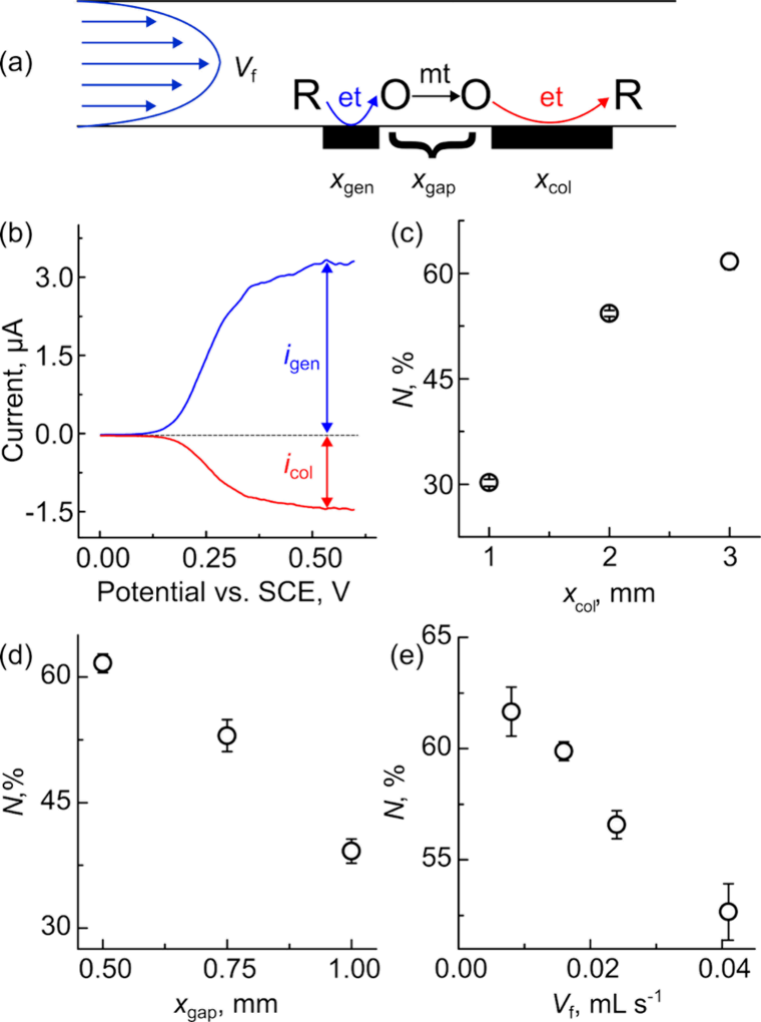
3D-printed dual-electrode generator–collector electrodes are easily fabricated using single-step multi-material 3D printing and show collection efficiencies greater than 60%. (a) Schematic of generator–collector experiment, where an upstream electrode generates oxidizes the reactant (O) to product (R), the convection within the channel transports R to the collector electrode, where it regenerates O. (b) Representative generation collection experiment where FcMeOH is oxidized on the generator electrode and FcMeOH^+^ is reduced on the collector electrode. The impact of (c) collector electrode length and (d) gap between electrodes on the collection efficiency. (e) Impact of *V*_f_ on the collection efficiency of a device with a 0.5 mm gap and 3 mm collector. The voltammetric data was collected using a 1 mM solution of FcMeOH in 0.1 M KNO_3_ at 0.05 V s^–1^. The flow rate in parts (b)–(d) was 0.016 mL s^–1^.

We first confirmed the hydrodynamics behaved according to the Levich equation for a CFE on both generator and collector electrodes by performing independent hydrodynamic calibrations of each electrode over the range of flow rates from 0–0.05 mL s^–1^ (Figure S12). Similar to the mixing device above and our previous channel-flow cells,^15^ we observed a strong correlation between *i*_lim_ and *V*_f_^1/3^ for each electrode (*R* ^2^_gen_ > 0.9999 and *R* ^2^_col_ = 0.9904), indicative of laminar, parabolic flow in a channel system.

Figure 4b shows typical LSVs for a dual-electrode generator–collector experiment. In these experiments, the potential of the generator electrode (blue trace) is swept from 0 to 0.6 V at a scan rate of 0.05 V s^–1^ to oxidize FcMeOH to FcMeOH^+^. The generator LSV shows the transport limited oxidation of FcMeOH, with a well-defined LSV that reaches a steady state around ∼0.5 V. Some variation in the current is observed as the voltammogram becomes controlled by mass transport (∼0.25 V), which is likely due to pump noise or fluid velocity oscillations caused by surface roughness. After being oxidized, the FcMeOH^+^ is carried to the collector electrode, held at a constant potential of 0 V, where it is reduced to FcMeOH at a transport limited rate (Figure 4b, red trace). In the absence of flow, the generator electrode displays a peak-shaped, quasi-reversible LSV, while the collector electrode measures no faradaic current.

The collection efficiency, *N*, describes the efficiency of the collector electrode to reduce the oxidized product from the generator electrode:

$$N=-\frac{i_{\lim}^{col}}{i_{\lim}^{gen}}\times 100\%$$ 5

where *i*_*lim*_^*col*^ is the limiting current of the collector electrode and *i*_*lim*_^*gen*^ is the limiting current of the generator electrode. One advantage of channel-based generator–collector systems is the ability to probe *N* over various transport regimes, which impact the collection efficiency. Several recent papers have shown that the collection efficiencies in DCE devices typically surpass those attained in RDE systems,^30,47,45,46^ which are typically around 20-30%.^39^

We expected that *N* would depend strongly on the geometry of the device, based on predictions from theory^41^ and previous experiments.^30,47,45,46^ We prepared a series of devices to systematically explore how the spacing between the two electrodes, *x*_gap_, and the length of the collector electrode, *x*_col_, affected *N*. We fixed the length of the upstream generator electrode, *x*_gen_, to be 0.5 mm, which was the smallest size we could reliably print in our setup (Figure S4). For each device, we characterized the limiting current as a function of flow rate to ensure the hydrodynamics within the cell were consistent with the channel flow geometry. We also performed generation/collection experiments over the *V*_f_ range from 0–0.05 mL s^–1^ to determine how the flow rate affected the collection efficiency.

First, we characterized devices with a 0.5 mm gap, and 1, 2, and 3 mm collector lengths. The experiments employed the oxidation of 1 mM FcMeOH in 0.1 M KNO_3_ at a scan rate of 0.05 V s^–1^ and flow rate of 0.016 mL s^–1^. Figure 4c shows that as the collector length increases from 1 to 3 mm, the collection efficiency increases from 30.1 ± 0.5% to 61.6 ± 1.1%. Next, we investigated the gap length between a 0.5 mm generator electrode and a 3 mm collector electrode. Figure 4d shows that as the gap between the electrodes increases from 0.5 to 1.0 mm, the collection efficiency decreases from 62 ± 1% to 39 ± 1%. Figure 4e shows the effect of volumetric flow rate on *N* for a device with a 0.5 mm generator electrode, 0.5 mm gap, and a 3 mm collector electrode. As the flow rate increases from 0.008 to 0.041 mL s^–1^, the collection efficiency decreases from 62 ± 1 to 52.7 ± 1.3%. Given that the collection efficiency varies with flow rate, it is likely that the system is behaving in a thin layer regime.^48^ Crooks and co-workers observed dramatic improvements to *N* when the flow operated in a similar regime in microfabricated devices.^30^ Taken together, the data in Figure 4c–4e highlight the importance of the product transit time on the collection efficiency. The results are consistent with previous theoretical and experimental results, which show that the highest collection efficiencies are expected with small generators and large collectors separated by a small gap.^47,50^ When the gap between the electrodes is large, *N* decreases because the generated FcMeOH^+^ has more time to diffuse away into the channel. However, using a smaller gap ensures that less FcMeOH^+^ diffuses away. When the collector electrode is short, FcMeOH^+^ has less time to interact with the collector electrode and therefore fewer of the FcMeOH^+^ molecules can be reduced. With a longer collector electrode, the time required for diffusion to the electrode increases and the amount of FcMeOH^+^ reduced also increases. Accordingly, the collection efficiencies are highest when *V*_f_ is low because at higher flow rates, FcMeOH^+^ is carried downstream before it can be reduced at the collector.

The collection efficiencies of dual-electrode generator–collector electrodes prepared using 3D printing compare extremely well to those developed using more elaborate, expensive, and complex techniques such as microfluidics. While the Crooks group reported collection efficiencies of close to 100% for low-flow conditions in microfluidic devices,^30,45^ typical collection efficiencies are in the 30-50% range.^50^ However, Unwin and co-workers^47^ reported collection efficiencies of ∼60% using a microfluidic device with *x*_gen_ = 25 μm, *x*_gap_ = 25 μm, and *x*_col_ = 400 μm. Earlier, Compton and Stearn showed collection efficiencies of ∼40% using devices with *x*_gen_ = 2.33 mm, *x*_gap_ = 0.8 mm, and *x*_col_ = 1.42 mm at similar flow rates to those employed here.^48^ Moreover, there is scope for improving the collection efficiency by decreasing the flow rates further and lengthening or adjusting the geometry of the collector electrode. While shortening the gap between electrodes would also improve collection efficiency, we are unable to decrease *x*_gap_ because of the resolution of our 3D printer.

## Summary and Conclusions

In this work, we expanded the design and functionality of hydrodynamic electrochemical devices fabricated using single-step, multi-material 3D printing. By designing, fabricating, and characterizing impinging jet electrodes, serpentine mixing/dilution circuits, and dual-electrode generator–collector flow cells, we demonstrated that complex electrochemical geometries can be realized using commercially available 3D printers without post-assembly or bonding steps. Importantly, the approach described herein enables the fabrication of monolithic devices that do not require alignment, clamping, or assembly, which we hope will increase the accessibility of these widely used devices to broader communities.

Each device exhibited electrochemical responses consistent with established hydrodynamic theory for its respective geometry. The impinging jet electrodes achieved high mass transfer coefficients and displayed limiting currents that followed Levich behavior across the tested flow rate range. The 3D-printed mixing circuit enabled accurate, quantitative dilution up to 100-fold, with results that closely matched those obtained using traditional volumetric glassware. Integration of in-channel electrodes allowed real-time, on-chip detection of analytes, further validating the reliability of the device for analytical applications. Finally, the dual-electrode generator–collector cells exhibited high collection efficiencies comparable to or exceeding conventional systems prepared using complex, expensive methodologies.

Compared to conventional fluidic fabrication methods such as soft lithography, laser-scribed polymers, and laminated/machined microfluidic devices, the single-step multi-material 3D-printing strategy described herein offers an accessible route to high performance electrochemical flow-cell fabrication. Traditional methods provide higher spatial resolution compared with FDM 3D printing but require multi-step processing, bonding, and manual electrode integration, which increases fabrication time and device-to-device variability. In the case of soft lithography, a clean room is also required when preparing small (<100 μm) channels. In contrast, this approach enables the monolithic fabrication of flow channels and electrodes, with complex three-dimensional internal structures, including voids, in a single, alignment-free print, eliminating assembly and sealing steps while still achieving hydrodynamic and electrochemical performance comparable to microfabricated systems. While the devices described here have obvious advantages, two main trade-offs exist for their use: (i) the electrodes cannot be removed from the cell and polished or cleaned; (ii) the electrodes and flow channels cannot be directly characterized with secondary techniques (*e.g.*, SEM, Raman, etc.) unless the devices are destroyed. Moreover, it is very challenging to measure the precise geometry of the sealed channel and therefore we do not rigorously understand how surface roughness and other non-idealities from the 3D printing process impact the hydrodynamic electrochemistry.

Collectively, these results underscore the capability of 3D printing to produce integrated, high-performance electrochemical systems with hydrodynamic control. The ability to fabricate devices with complex internal features in a single, accessible printing step lowers the barrier to custom electrochemical cell design and promotes reproducibility through the sharing of printable .stl files. This approach offers a versatile platform for future developments in electroanalysis, electrocatalysis, and micro-/millifluidic electrochemical sensing, where rapid prototyping and modularity are essential.

## Supplementary Material





## Notes

(1) Schematics and images of flow systems and experimental setups, (2) comparison of mass transfer coefficients, (3) additional characterization of 3D-printed wall-jet electrodes, (4) additional characterization of 3D-printed mixing/dilution circuit with built-in electrodes, and (5) additional characterization of dual-electrode generator–collector cells (PDF)
.stl models of (i) wall-jet electrode, (ii) mixing/dilution device, and (iii) dual-electrode generator–collector cell (ZIP)
