# Supplementary material for: Expanding the Toolbox of Multi-Material Three-Dimensional-Printed Electrochemical Flow Cells Fabricated in a Single Step: Impinging Jet, Mixing and Dilution, and Dual-Electrode Generator–Collector Electrochemical Cells
Source: ACS Electrochem. 2026 Jan 30;2(3):651–60. doi: 10.1021/acselectrochem.5c00444 (PMC12969262; doi:10.1021/acselectrochem.5c00444)
Supplement: Supplementary file 1 [file ec5c00444_si_001.pdf]

Expanding the toolbox of multi-material 3D-printed electrochemical flow cells fabricated in a single step: impinging jet, mixing and dilu-tion, and dual-electrode generator-collector electrochemical cells

Kayla M. Mancini,[†1] Enock G. Arthur,[†1] Cameron Darvish,[†] Edgar M. Manriquez,[†] Inara Trongone,[†] and Glen D. O'Neil[†‡*]

[†]Department of Chemistry and Biochemistry, Montclair State University, Montclair, NJ 07043

[‡]Sokol Institute for Pharmaceutical Life Sciences, Montclair State University, Montclair, NJ 07043

[1] These authors contributed equally

*Email: oneilg@montclair.edu; Phone: +1 973-655-3791

Table of contents

Section **Page Number**

S1. Characterization of printed components……………………………………………………………S2

S2. Schematics and images of flow systems and experimental setups……………………………………S3

S3. Comparison of mass transfer coefficients……………………………………………………………. S6

S4. Additional characterization of 3D-printed wall-jet electrodes……………………………………….. S8

S5. Additional characterization of 3D-printed mixing/dilution circuit with built-in electrodes…………..
S9

S6. Additional characterization of dual-electrode generator-collector cells……………………………..S10

Section S1. Characterization of printed components

The dimensions of 3D-printed parts do not always agree with the dimensions set by the design file. The devices described here offer numerous challenges with characterizing the internal geometry (including surface roughness, the interface between the electrode and the insulator, and the channel height) of the channels and electrodes because they are monolithic. Therefore, typical characterizations like SEM, profilometry, even optical microscopy are not suitable for characterizing the *internal* geometry of the channels.

In an effort to provide some understanding of how the designed dimensions correlate to the printed dimensions, we cleaved the electrodes at ~90° to the channel using a saw, filled the channel with a mixture of black nail varnish and epoxy, and imaged the channel height and width on a microscope. We performed these experiments with a series of devices with designed widths over the range from 0.6 to 4.0 mm and heights from 0.35 to 1.00 mm.

Figure S1 compares the measured width (Figure S1a) and height (Figure S1b) to the designed height. The figure shows a clear linear correlation between the measured and designed dimensions. The linear regression lines are shown below each graph for clarity. For all but the smallest widths (0.06 cm), the error is ~20% (Table S1).

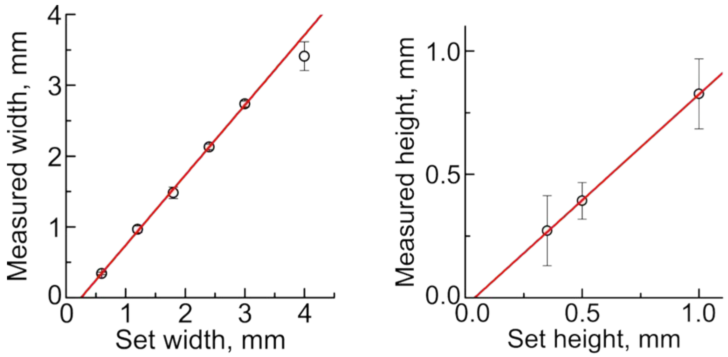

y = 0.99 ± 0.01x - 0.25 ± 0.02 R^2 = 0.9992

y = 0.86 ± 0.01x - 0.034 ± 0.009 R^2 = 0.9992

Figure S1. Comparison of measured and designed widths (a) and heights (b).

Table S1. Comparison of designed and measured widths and heights.

**Designed width, cm**	**Measured width, cm**	**Error, %**	**Designed height, cm**	**Measured height, cm**	**Error, %**
0.06	0.034 ± 0.002	43	0.035	0.027 ± 0.014	22
0.12	0.096 ± 0.005	20	0.05	0.039 ± 0.007	21
0.18	0.148 ± 0.008	18	0.1	0.083 ± 0.014	17
0.24	0.213 ± 0.004	11			
0.3	0.274 ± 0.004	9			
0.4	0.34 ± 0.02	15			

Section S2. Schematics and images of flow systems and experimental setups

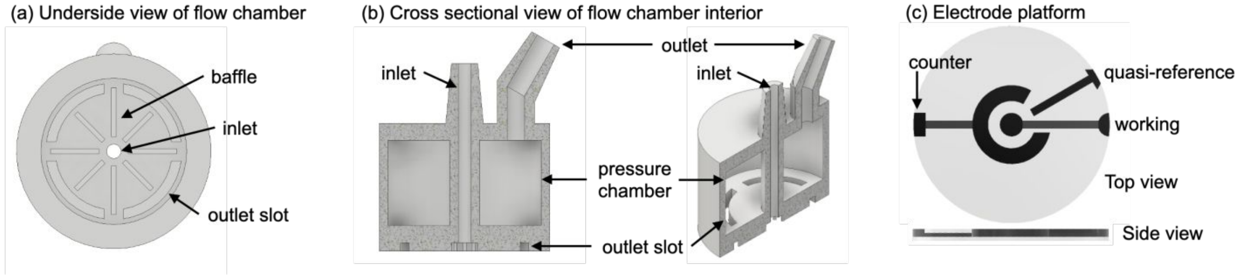

Figure S2. Schematic of impinging jet electrodes showing the (a) baffle layout, (b) pressure equilibration chamber, and (c) electrode configuration.

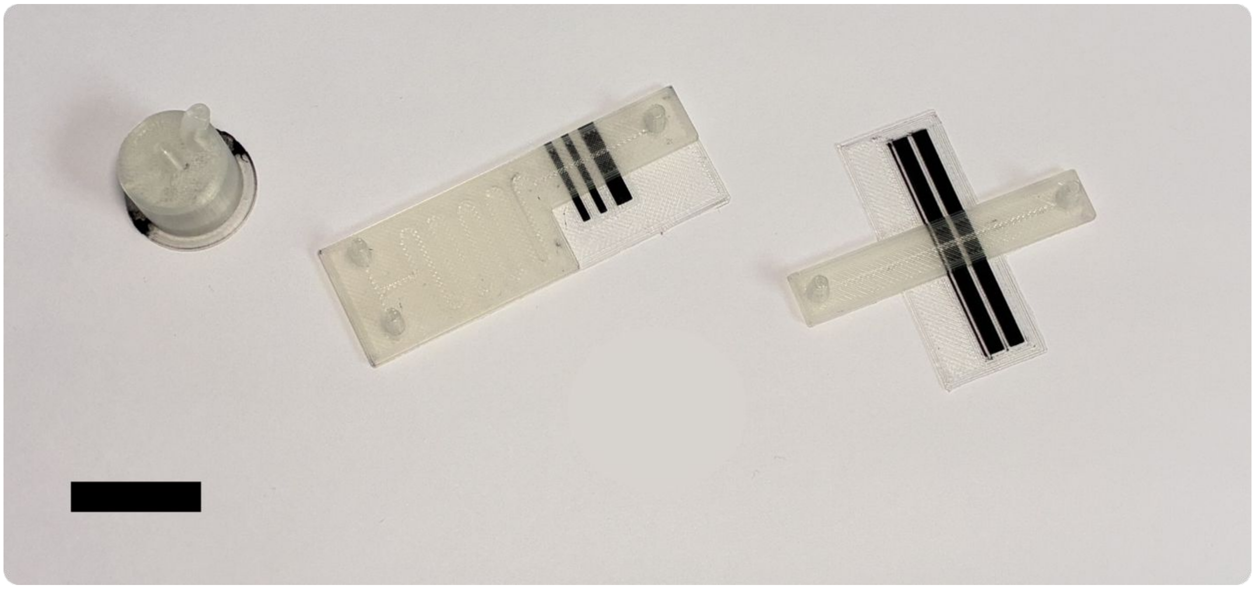

Figure S3. Photographs of the printed (a) impinging jet electrode, (b) mixing and dilution circuit with built in electrochemical detection, (c) dual-electrode generator-collector cell. Scale bar is 2.54 cm.

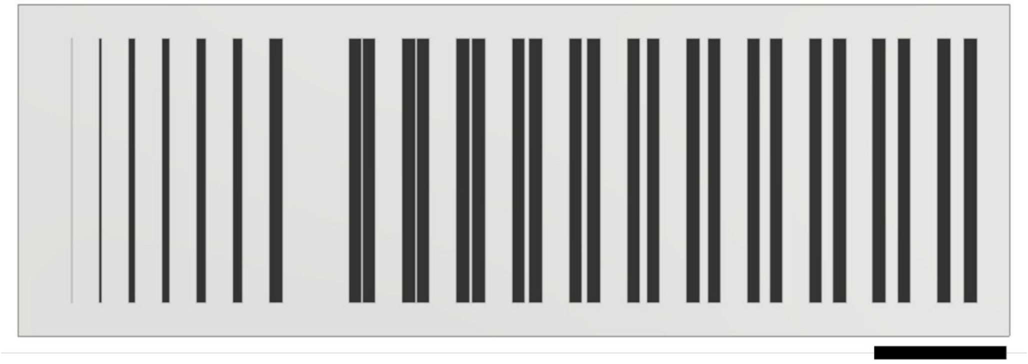

Figure S4. CAD rendered schematic of the device used to assess 3D printing resolution. Scale bar = 1 cm.

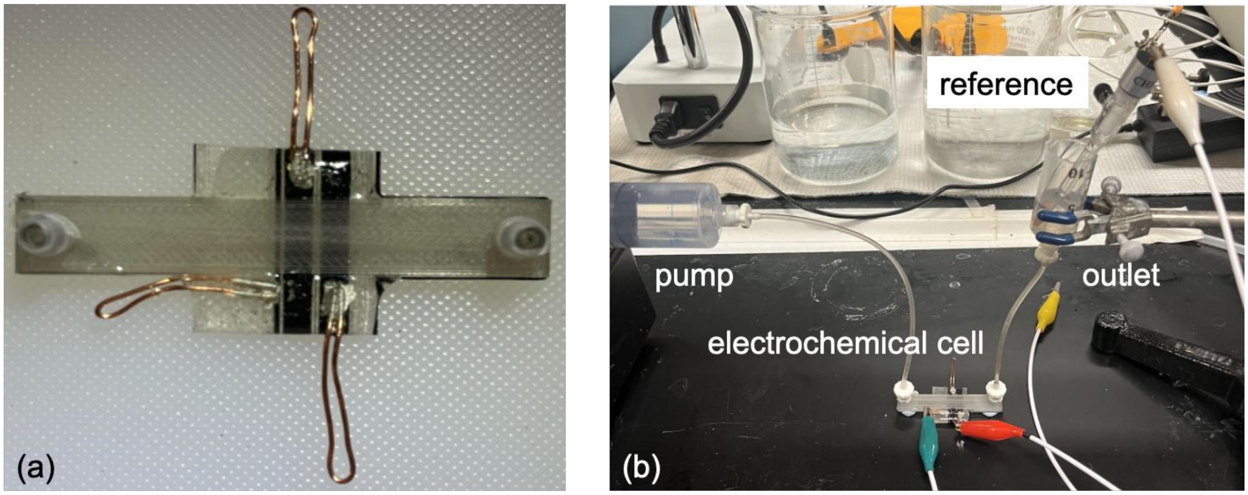

Figure S5. Photographs of experimental setup used for hydrodynamic electrochemistry.

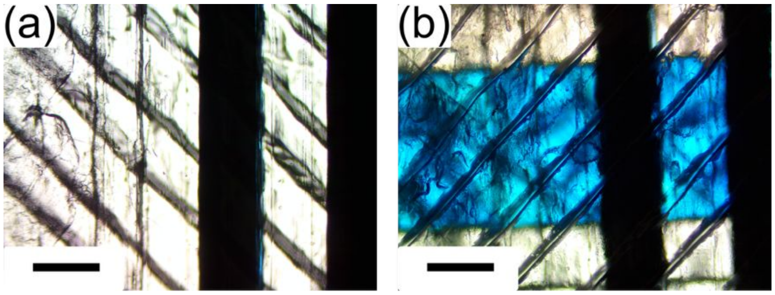

Figure S6. Microscope images of the dual-electrode generator-collector electrodes. The channel was filled with an aqueous methylene blue solution to aid visualization of the channel. (a) Image collected from the underside of the device in transmission mode; (b) image collected from the top side of the device outside of the channel to visualize the electrodes. Scale bar = 0.5 mm.

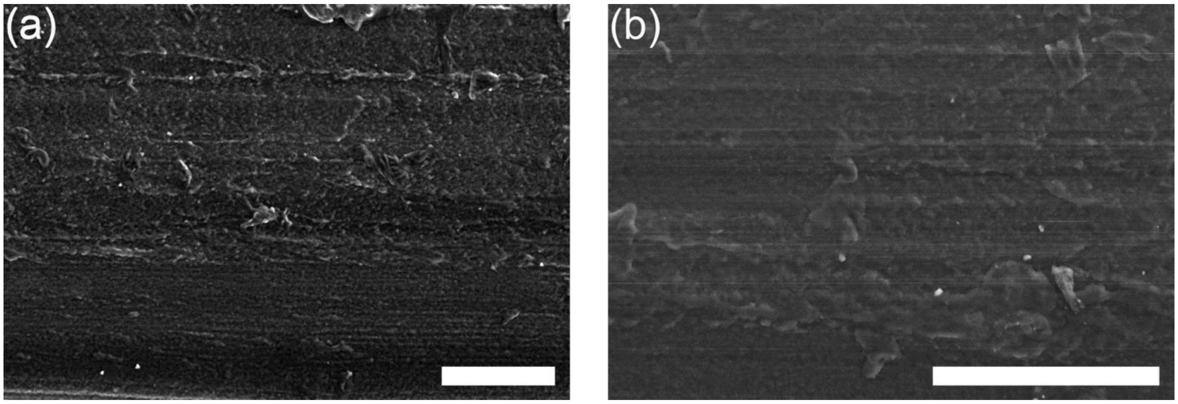

Figure S7. SEM micrographs of as-printed carbon black/PLA electrodes. Note that the samples for these images were printed under the same conditions as the devices described in the main text, but did not include a flow channel above the electrode. Scale bar = 100 µm.

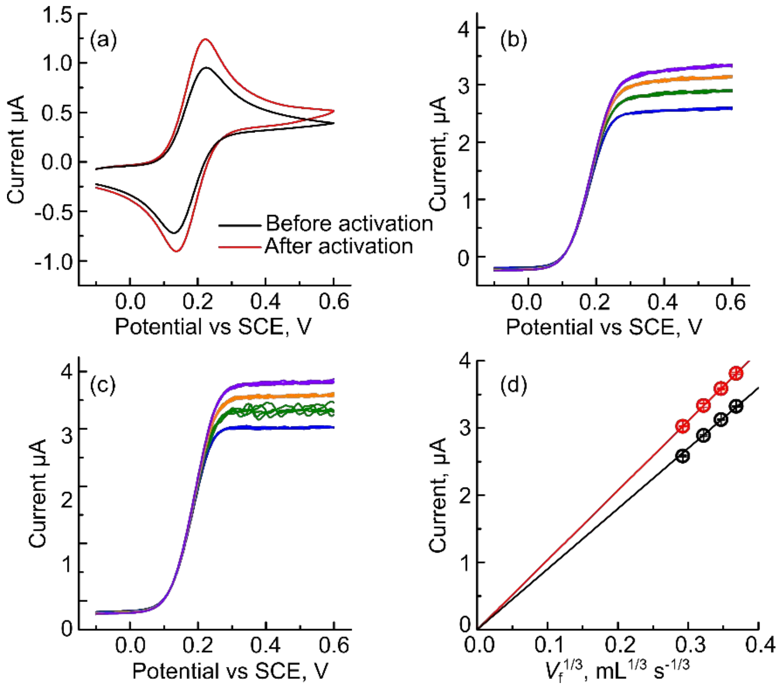

Figure S8. The effect of activation of monolithic flow cell electrodes using 0.5 M NaOH. (a) stationary (V_f = 0 mL s^{-1}) CV of a channel flow electrode using 0.5 mM FcMeOH in 0.1 M KNO_3 at 0.1 V s^{-1}; Five LSVs of the oxidation of 1 mM FcMeOH in 0.1 M KNO_3 at 0.05 V s^{-1} at flow rates ranging from 0.025 (blue trace) to 0.5 (purple trace) mL s^{-1} (b) before and (c) after activation with 0.5 M NaOH; (d) (c) i_{lim} versus $V_f^{3/4}$ for the data shown in parts (b) and (c).

Section S3. Comparison of mass transfer coefficients

To estimate the enhancement in mass transfer expected for wall-jet electrodes compared with channel flow electrodes, we first present the equations that describe the mass transfer coefficient, k_t, for the two systems employed here. For comparison, we also show the relevant expressions for rotating disk electrodes (RDEs) and ultramicroelectrodes (UMEs). Finally, we note that the geometry of CFEs, WJEs, and UMEs impacts the k_t. In order to make reasonable comparisons for the CFEs and WJEs, we compared geometries that were used in this work and that we've found are printable with high success rates. We used a UME radius of 12.5 μm because that corresponds to commercial Pt UMEs that are readily available. Finally, we employed a 100 Hz rotation rate that is achievable for most commercial RDE setups.

In Equations S1-S9, the following variables are used: i_{lim} is the limiting current (in A), n is the number of electrons transferred, F is Faraday's constant (=96485 C mol^{-1}), A is the electrode area (in cm^2), c^* is the bulk concentration of redox mediator (in mol cm^{-3}), D is the diffusion coefficient (in cm^2 s^{-1}), V_f is the volumetric flow rate (in cm^3 s^{-1}), w is the CFE channel width (in cm), h is ½ the CFE channel height (in cm), x_e is the electrode length (in cm), k_c is a momentum flux constant (= 0.9), $r_{\text{electrode}}$ is the electrode radius, r_{inlet} is the inlet radius, v is the kinematic viscosity (= 8.8•10^{-3} cm^2 s^{-1}), and f is the frequency (in s^{-1}).

In general, k_t is described by:

$$k_t = \frac{i_{lim}}{nFAc^*} \quad \text{(S1)}$$

For a channel flow electrode, the limiting current is given by:

$$i_{lim} = \frac{0.925nFc^*D^{2/3}V_f^{1/3}w^{2/3}x_e^{2/3}}{h^{2/3}} \quad \text{(S2)}$$

And k_t is given by, assuming that the area of the electrode is equal to the product of channel width and electrode length:

$$k_t = \frac{0.925nFc^*D^{2/3}V_f^{1/3}}{w^{1/3}x_e^{1/3}h^{2/3}} \quad \text{(S3)}$$

For a wall-jet electrode, the limiting current is given by:

$$\frac{i_{lim}=1.597nFk_c r_{electrode}^{3/4}D^{2/3}V_f^{3/4}c^*}{v^{5/12}r_{inlet}^{1/2}} \quad \text{(S4)}$$

And k_t is given by:

$$k_t = \frac{0.458D^{2/3}V_f^{3/4}}{v^{5/12}r_{inlet}^{1/2}r_{electrode}^{5/4}} \quad \text{(S5)}$$

For an RDE, the limiting current is given by:

$$i_{lim} = \frac{1.554nFAD^{2/3}f^{1/2}C^*}{v^{1/6}} \quad \text{(S6)}$$

And kt is given by:

$$k_t = \frac{1.554D^{2/3}f^{1/2}}{v^{1/6}} \quad \text{(S7)}$$

Finally, the limiting current for a disk shaped ultramicroelectrode under diffusion control is given by:

$$i_{lim} = 4nFDC^*r_{electrode} \quad \text{(S8)}$$

And k_t is given by:

$$k_t = \frac{4D}{\pi r_{electrode}} \quad \text{(S9)}$$

Table S1 shows a comparison of k_t values for the systems employed here (note the details of device geometry are included in the Table). In the comparative estimates, $D = 7.8\times10^{-6}$ cm^2 s^{-1} and $v = 8.8\times10^{-3}$ cm^2 s^{-1}. As shown in the table, the RDE shows the fastest rates of mass transfer (=0.013 cm s^{-1}) due to the high rotation rates available with commercial instrumentation (6000 rpm). The WJE has a similar mass transfer rate to the UME. Finally, the CFE has the slowest mass transfer rate.

Table S2. Comparison of mass transfer coefficients of various hydrodynamic electrochemical cells.

**Cell geometry**	**k_t, cm s^{-1}**
CFE[a,b]	0.00026
CFE[a,c]	0.00044
WJE[b,d]	0.0051
WJE[c,d]	0.017
RDE[e]	0.013
UME[f]	0.0080

[a] Dimensions of cell: $w = 0.47$ cm, $2h = 0.1$ cm, $x_e = 0.15$ cm; [b] $V_f = 0.05$ mL s^{-1}; [d] Dimensions of cell: $r_{electrode} = 0.15$ cm, $r_{inlet} = 0.075$ cm; [e] $f = 100$ Hz / 6000 rpm; [f] $r_{electrode} = 12.5$ µm.

Section S4. Additional characterization of 3D-printed wall-jet electrodes.

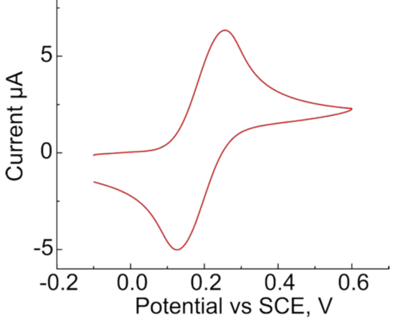

Figure S9. Stationary CV of 0.5 mM FcMeOH in 0.1 M KNO_3 for a wall-jet electrode with $r_{electrode} = 0.15$ cm collected at 0.05 V s^{-1}.

We compared the experimental peak current of the wall-jet electrodes to those calculated using the Randles-Ševčík equation for quasi-reversible electron transfer at 25 ºC:

$$i_p = 2.63 \bullet 10^5 n^{3/2} A D^{1/2} c_b v^{1/2} \quad \text{(S10)}$$

where n is the number of electrons transferred (= 1), A is the electrode area (= 0.0707 cm^2; from πr^2), D is the diffusion coefficient (= $7.8 \bullet 10^{-6}$ cm^2 s^{-1}), c_b is the concentration of FcMeOH (=$5 \bullet 10^{-7}$ mol cm^{-3}), v is the scan rate (0.05 V s^{-1}).[86] Note that the numerical constant in quasi-reversible form of the Randles-Ševčík equation (= $2.63 \bullet 10^5$) is slightly smaller than the reversible form (= $2.69 \bullet 10^5$).

Our syringe pumps are limited to flow rates of ~180 mL s^{-1} (3 mL min^{-1}). In order to test our WJE at higher flow rates, we employed a gravity fed pump, featuring a 500 mL reservoir positioned ~1.4 m above the lab bench. The reservoir was connected to the flow cell using ¼" inner diameter tubing. We crudely estimated the flow rate by dispensing solution for 30 s through the flow cell into a graduated cylinder and recording the volume. The flow rate was stable over several minutes, as judged by repetitive LSVs under flowing conditions. These experiments are not quantitative and aim to show the dramatic improvements in current observed with increased flow rates.

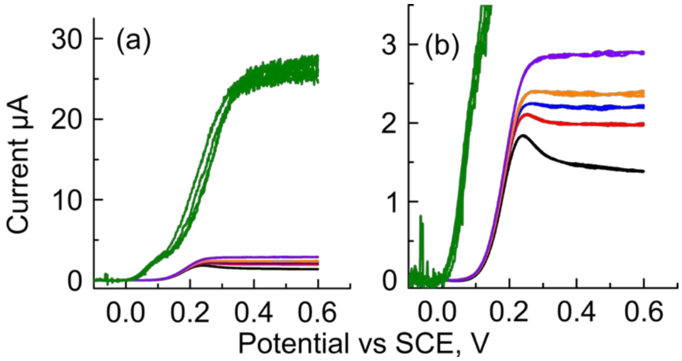

Figure S10. LSVs (n = 3 for each flow rate) of the oxidation of 0.5 mM FcMeOH in 0.1 M KNO_3 using an impinging jet electrode with r_{inlet} = 2 mm and $r_{electrode}$ = 2 mm tested over flow rates from 0.017 to 0.05 mL s^{-1} (black through purple traces) delivered with a syringe pump and at ~4 mL s^{-1} using a gravity-fed pump. (a) shows all of the flow rates and (b) shows a magnified view of the lower flow rates. The lower flow rates used a SCE as the reference electrode while the gravity-fed flow measurements used a 3D-printed on-device reference.

Section S5. Additional characterization of 3D-printed mixing/dilution circuit with built-in electrodes.

Table S3. Determination of $Fe(CN)_6^{3-}$ from the 3D printed fluidic mixer device.

**Dilution fraction**	V_f **(stock), mL s⁻¹**	V_f **(diluent), mL s⁻¹**	**[$Fe(CN)_6^{3-}$], mM**
1	0.0160	0.0	2.987
0.9	0.0144	0.0016	2.688
0.75	0.0120	0.0040	2.240
0.5	0.0080	0.0080	1.493
0.25	0.0040	0.0120	0.747
0.1	0.0016	0.0144	0.299
0.075	0.0012	0.0148	0.224
0.05	0.0008	0.0152	0.149
0.025	0.0004	0.0156	0.075
0.01	0.00016	0.01584	0.030

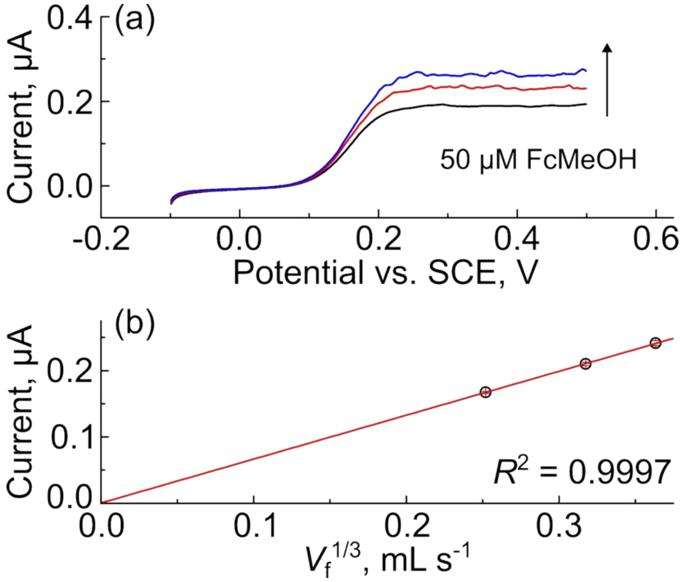

Figure S11. Hydrodynamic characterization of mixing/dilution cell. (a) LSVs collected at 0.016, 0.032, and 0.048 mL s^{-1} in a solution of 50 µM FcMeOH also containing 0.1 M KNO_3. The scan rate was 0.025 V s^{-1}. (b) Plot of the background subtracted limiting currents versus the $V_f^{1/3}$ showing a linear relationship, confirming laminar flow within the devices.

Section S6. Additional characterization of dual-electrode generator-collector cells.

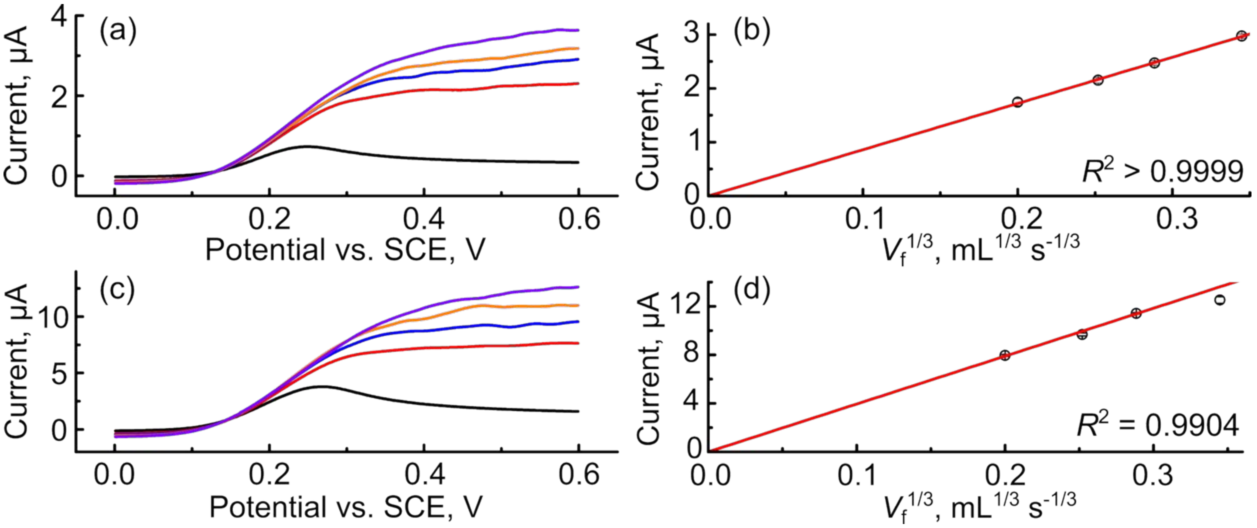

Figure S12. Hydrodynamic characterization of generator-collector cell shows that mass transport is consistent with parabolic flow in a microchannel. (a) Average LSVs (n = 5; one device) for the generator electrode (x_{gen} = 0.5 mm); (b) plot of the limiting current versus $V_f^{1/3}$ for the device shown in (a); (c) Average LSVs (n = 5; one device) for the collector electrode (x_{gen} = 0.5 mm); (d) plot of the limiting current versus $V_f^{1/3}$ for the device shown in (c). The best fit line in (d) omits the data collected at the highest flow rate.
